# Sentinel node detection in endometrial cancer using indocyanine green and fluorescence imaging—a case report

**DOI:** 10.3332/ecancer.2015.549

**Published:** 2015-06-29

**Authors:** Anupama Rajanbabu, R Venkatesan, Satish Chandramouli, P V Nitu

**Affiliations:** Amrita institute of Medical Sciences, Amrita Vishwa Vidyapeetham, Kochi, Kerala 682041, India

**Keywords:** endometrial cancer, sentinel node, fluorescent lymphoscintigraphy

## Abstract

Sentinel lymph node mapping in endometrial cancer can help to provide the prognostic information needed while avoiding the morbidity associated with a complete lymphadenectomy. Studies with blue dye and technetium colloid have only given about 80% detection rates whereas with indocyanine green injection and fluorescence imaging, it gives about 88–100% detection rates. Herein, we report a case where indocyanine green was injected intracervically and sentinel nodes were detected at the paraaortic nodal area.

## Introduction

Lymphadenectomy in endometrial cancer is a topic of ongoing debate. The presence of lymph node involvement is one of the most important prognostic factors in endometrial cancer, but the therapeutic benefit of lymphadenectomy is doubtful. The technique of sentinel lymph node (SLN) mapping has relevance in this scenario as it can provide the prognostic information needed while avoiding the morbidity associated with a complete lymphadenectomy [1]. Many studies have come up with cervical, subserosal, and hysteroscopic injections using blue dye and technetium-99 colloid, but this has detection rates of less than 80% [1, 2]. The use of near infrared fluorescence imaging after intracervical injection with indocyanine green (ICG) dye has given 87–100% detection rates and hence appears superior to the use of blue dye or radioactive colloid [3–7]. We report a case of SLN mapping using ICG and fluorescence imaging in endometrial cancer which is the first case carried out in India.

## Case report

A 57-year-old lady reported to our institution with an endometrial biopsy showing well-differentiated adenocarcinoma. Slide review confirmed the diagnosis. Magnetic resonance imaging (MRI) of the pelvis showed a 3 × 2 cm lesion in the endometrial cavity with superficial myometrial invasion without any enlarged pelvic or paraaortic nodes. She underwent robotic staging surgery for carcinoma endometrium in February 2015 using the da Vinci Xi robotic platform. As there is facility for fluorescence imaging in the robotic platform, it was decided to use ICG for sentinel mapping in her. Consent for the procedure was obtained from the patient and also from the hospital’s ethical committee.

**Figure 1.**
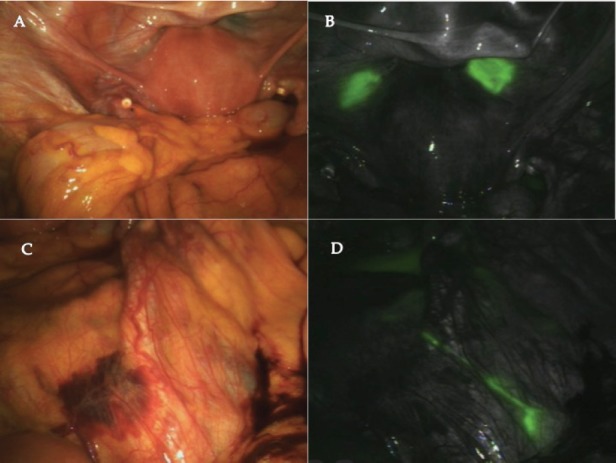
A) Appearance of pelvic area under normal light. B) Pelvic area with fluorescent imaging showing lighting up of the injection site. No lymphatics are seen going to the pelvic nodes. C) Paraaortic area seen under normal light. D) Paraaortic area with fluorescent imaging showing the lymphatic crossing right common iliac artery near the bifurcation and going to the right periaortic node just below the duodenum.

Concentration of ICG used was 0.5 mg/mL, a 4 mL of this solution was injected intracervically at 3 and 9 o’clock positions, both submucosally and deep into the cervical stroma. The injection was done after port placement but before the docking of the robotic platform. After complete inspection of peritoneal cavity and collecting pelvic washings, the fluorescent imaging was switched on. The time taken from the cervical injection till this point was 20 minutes. Under fluorescence imaging, the site of intracervical injection was well lit up but no lymphatics or lymph nodes were identified in the pelvic nodal areas. We then looked into the paraaortic area with fluorescence imaging and saw a lit up lymphatic channel crossing the right common iliac and lighting up of a right periaortic node (Figure 1). Bilateral pelvic lymphadenectomy and removal of the lighted up paraaortic nodes were done. Complete paraaortic nodal dissection was not done as it is our practice to do paraaortic lymphadenectomy for high-risk histologies.

Histopathology report showed 4.5 × 4 × 2.5 cm grade 2 endometrioid carcinoma infiltrating to outer one-half of the myometrium with lymphovascular emboli. Two out of three paraaortic nodes removed were positive for tumour metastasis. Three out of 11 left iliac nodes were also found to be positive, and the ten right iliac nodes removed were found to be negative. The case was discussed by the multidisciplinary tumour board, and it was decided to give her adjuvant radiotherapy with chemotherapy in view of node positivity.

## Conclusion

The use of SLN mapping in endometrial cancer is gaining in acceptance [4–7]. A metaanalysis involving 26 studies using blue dye of radioactive colloid have shown a 78% detection rate for SLN mapping in endometrial cancer which is lower than that for melanoma, breast cancer, or colorectal cancer [2]. Intracervical injection of ICG followed by fluorescent imaging appears to be a simpler technique with improved detection rates [4]. We had tried sentinel node detection for endometrial cancer using blue dye and technetium previously, but the detection of the nodes was very difficult. This was our first experience with ICG injection followed by fluorescent imaging and the technique appeared simple providing clear view of the sentinel node. More prospective studies are needed to validate the accuracy of this method.
